# Child-caregiver interaction in two remote Indigenous Australian communities

**DOI:** 10.3389/fpsyg.2015.00514

**Published:** 2015-04-29

**Authors:** Jill Vaughan, Gillian Wigglesworth, Deborah Loakes, Samantha Disbray, Karin Moses

**Affiliations:** ^1^School of Languages and Linguistics, University of MelbourneParkville, VIC, Australia; ^2^Northern Institute, Charles Darwin UniversityAlice Springs, NT, Australia; ^3^Student Learning, La Trobe UniversityBendigo, VIC, Australia

**Keywords:** child language acquisition, language input, language shift, Walmajarri, Fitzroy Valley Kriol, Warumungu, Wumpurrarni English

## Abstract

This paper reports on a study in two remote multilingual Indigenous Australian communities: Yakanarra in the Kimberley region of Western Australia and Tennant Creek in the Barkly region of the Northern Territory. In both communities, processes of language shift are underway from a traditional language (Walmajarri and Warumungu, respectively) to a local creole variety (Fitzroy Valley Kriol and Wumpurrarni English, respectively). The study focuses on language input from primary caregivers to a group of preschool children, and on the children's productive language. The study further highlights child-caregiver interactions as a site of importance in understanding the broader processes of language shift. We use longitudinal data from two time-points, approximately 2 years apart, to explore changes in adult input over time and developmental patterns in the children's speech. At both time points, the local creole varieties are the preferred codes of communication for the dyads in this study, although there is some use of the traditional language in both communities. Results show that for measures of turn length (MLT), there are notable differences between the two communities for both the focus children and their caregivers. In Tennant Creek, children and caregivers use longer turns at Time 2, while in Yakanarra the picture is more variable. The two communities also show differing trends in terms of conversational load (MLT ratio). For measures of morphosyntactic complexity (MLU), children and caregivers in Tennant Creek use more complex utterances at Time 2, while caregivers in Yakanarra show less complexity in their language at that time point. The study's findings contribute to providing a more detailed picture of the multilingual practices at Yakanarra and Tennant Creek, with implications for understanding broader processes of language shift. They also elucidate how children's language and linguistic input varies diachronically across time. As such, we contribute to understandings of normative language development for non-Western, non middle-class children in multilingual contexts.

## Introduction

Indigenous children in Australia grow up in a range of diverse language settings. In the cities and metropolitan areas, particularly along the populated coastlines of Australia, which were settled early by Europeans, traditional languages have long ceased to be spoken as the primary means of day-to-day communication. Instead, local varieties of Aboriginal English are spoken as in-community and in-family codes. “Aboriginal English” is an umbrella term, covering a range of varieties in different sociolinguistic and geographic settings (see Malcolm and Kaldor, 1991). Aboriginal English is largely mutually intelligible with standard Australian English, and in its “lightest” styles differs only in minor ways from the standard, usually in pronunciation, lexis, grammatical patterns, speech pragmatics and conceptualization (see e.g., Kaldor and Malcolm, 1982; Malcolm and Kaldor, 1991; Eades, 1996; Malcolm and Sharifian, 2002, 2007; McGregor, 2004; Sharifian, 2005; Butcher, 2008).

In more remote locations, particularly in central and northern Australia, the picture is more complex. In some places, traditional languages continue to be acquired as first languages by the current generation of children, and in other places, language shift to contact varieties, including mixed languages and local creole varieties, has taken place, or is in process (see e.g., McConvell and Thieberger, 2001). Creole varieties on mainland Australia are referred to as *Kriol*, the name being based on the word *creole*^1^. There are a number of locally named varieties of Kriol, for example in Yakanarra (one of our field sites), the variety is called Fitzroy Valley Kriol (or Kimberley Kriol) (see e.g., Hudson, 1985), henceforth FVKriol. Another example is Roper River Kriol spoken in the Ngukurr region. In Tennant Creek (our second field site) the creole is referred to as Wumpurrarni English, henceforth WE, and varies from acrolectal (more like English) to basilectal (a heavy creole) (see e.g., Disbray and Simpson, 2004; Disbray, 2009)^2^.

Recent research into such language settings in Australia is providing descriptions of children's language acquisition in diverse and dynamic contexts (see, for example, the variety of papers in Simpson and Wigglesworth, 2008). Other research focuses on the rapidly changing language environments and the emergence of new contact varieties (e.g., Bavin and Shopen, 1985 for Yuendumu Warlpiri; McConvell and Meakins, 2005; Meakins, 2011 for Gurindji Kriol; O'Shannessy, 2005 for Light Warlpiri and McConvell, 2008 for general discussion of mixed languages in Indigenous Australia).

This study is an investigation of language use amongst Indigenous caregivers and their young children in two remote communities in Australia. There are two themes to the paper. The first regards child language development, and the second compares how this manifests in two communities of Indigenous people that are in many ways comparable (remote, multiple codes spoken, community language endangerment, and shift) but are markedly different in terms of the sociolinguistic setting and the mixture of languages that children are exposed to.

Our own work has looked in some detail at the language situation in Indigenous Australia. The work is associated with The Aboriginal Child Language Acquisition (ACLA1) study^3^, carried out from 2004 to 2007, which focused on child-directed speech, as well as language use by children, in three Indigenous Australian communities (for more information on ACLA1 see Wigglesworth and Simpson, 2008)—Yakanarra, Tennant Creek and Kalkaringi^4^. This is particularly interesting because of the relative dearth of information on how non-Western children acquire language, and also because these particular children live in multilingual societies which are undergoing rapid language shift. We address prior research in this area further below.

### Studies of child-directed speech

We have looked, for example, at the way Yakanarra children deal with questions in an informal setting (Moses and Yallop, 2008), at the language codes children receive from different aged interlocutors (Loakes et al., 2013), and children's knowledge of their traditional language (Loakes et al., 2012). From this, we know that Indigenous children in Yakanarra frequently experience questions and respond to questioning, debunking earlier views that Indigenous Australians are not familiar with question-answer routines (Moses and Yallop, 2008). We know that children speak and hear FVKriol predominantly, but are also exposed to the traditional language Walmajarri, especially from older community members. As far as Walmajarri is concerned, we also know that children have relatively good receptive knowledge when it comes to nouns (Loakes et al., 2012), and we also know that their traditional language knowledge goes beyond this, because they may respond to Walmajarri utterances from an elder entirely (and appropriately) in FVKriol (examples shown in Loakes et al., 2013).

In Tennant Creek we have investigated interactions between caregivers and children during joint picture-book viewing (Disbray, 2008). In these interactions we saw that caregivers of the children aged between 18 months and 5 years of age were sensitive to the child's level of attention at different ages, and that through questioning and prompting, and through repetition and elaboration, they collaboratively “built” a rich narrative. This study also established common patterns of use of Warumungu features in WE, further explored as a factor of the age of the adult speaker in Morrison and Disbray (2008). These studies have shown that children hear full Warumungu only from elderly speakers, and that in the WE speech of younger adults, some common Warumungu nouns and fewer Warumungu verbs are used, a bilingual speech pattern which children also later develop (Disbray, 2009). Additionally, semantic case-markers, derived from Warumungu, have been incorporated into WE, and are used variably (Disbray and Simpson, 2004; Morrison and Disbray, 2008).

We know that, in general, children have acquired most of the skills needed for adult-like language proficiency by the time they attend primary school (Hoff, 2001), and spend their school years mastering more complex grammatical and phonological features. However, studies of the development of grammaticality in Indigenous Australian children have only recently gained momentum. Malcolm (1996) reports on development of the verb phrase in Aboriginal English spoken in a remote Indigenous region, and concludes that there is evidence that language development may be ongoing for bilingual/bidialectal Indigenous children even between 5 and 10 years of age. Disbray (2009) investigated the development of discourse competence among 5–14 year old WE-speaking children, demonstrating that the children pass through similar developmental stages to children speaking other languages to create a cohesive text. A great deal of further research is needed to understand language development amongst Indigenous Australian children.

Previous studies of child-directed speech have noted that parental speech to children can involve utterances that are shorter, morphosyntactically simpler, and more redundant than speech directed at adults (e.g., Snow, 1972, 1977; Sachs et al., 1976; Ferguson, 1977). These findings contributed to the idea that simplified language input may help children's language development (e.g., Snow, 1972), often referred to as the “motherese hypothesis.” In the 2–5 year old age range, as in the present study, language development is characterized by a move from simple two- and three-word combinations to the production of more grammatically sophisticated utterances, as children become skilled at using most of the requisite grammatical morphemes and begin to construct complex multiclause sentences (Hoff, 2001; Berko Gleason, 2005).

Snow (1972) observed that maternal speech to 2-year olds is simpler and more redundant than speech directed at 10-year-olds, and other studies focusing on younger children in the fundamental stages of language acquisition have demonstrated that caregivers make fine adjustments to the complexity of their speech according to the age of the child (e.g., Vosoughi, 2010). Kaye (1980), Philips (1973) and Rondal (1980) all observed markedly shorter, simpler, and more repetitive utterances to infants than to toddler-aged children. A more recent study by Huttenlocher et al. (2007) with children aged 14–30 months reinforces these early findings; their analysis of the speech used by English-speaking parents with their children at 4-month intervals revealed that caregivers made substantial adjustments to the syntactic complexity and diversity of their speech over the 16-month time period. In the sample of 50 parents, Huttenlocher et al. also found individual differences in input patterns related to caregiver education level, and noted that caregivers maintained their idiosyncratic input patterns at the same time as making incremental adjustments to their speech depending on the age of their child interlocutor. The researchers suggest that this consistent adjustment indicates caregiver sensitivity to the language levels of young children, while providing the children with a progressively broader set of language models.

Other studies incorporating child production data have demonstrated that longer and more complex utterances from caregivers correlate with more advanced morphosyntactic development in children. Hoff-Ginsberg (1998), in a study of maternal speech to children based on birth order, found that first-born children showed “more precocious lexical and grammatical development than later borns” (Hoff-Ginsberg, 1998, p. 626), and that this correlated with longer and more complex utterances from their mothers. Huttenlocher et al. (2002) looked at the production of multiclause utterances by 4-year olds in relation to parental input at home, and teacher input at pre-school, and found that the style of parental input was the major predictor of syntactic complexity in children's speech. In addition, various studies have shown the complexity, quantity, and lexical diversity of caregiver speech to have strong correlations with children's vocabulary development (Huttenlocher et al., 1991; Hart and Risley, 1995; Bornstein et al., 1998; Naigles and Hoff-Ginsberg, 1998; Hoff and Naigles, 2002; Hoff, 2003; Pan et al., 2005; Rowe, 2008).

Recent studies of the acquisition of sociolinguistic variation (e.g., Foulkes et al., 1999, 2005; Díaz-Campos, 2005; Smith et al., 2007, 2013; O'Shannessy, 2011) emphasize that child-directed speech plays a central role in socializing children into the appropriate use and the social-indexical values of variables (Foulkes et al., 2005, p. 177). For example, in their study of 29 child/caregiver pairs in a small Scottish fishing village, Smith et al. showed that patterns of variation are acquired at a very early age in tandem with language acquisition more generally, but that the nature of this process may vary according to the variable in question (2013, p. 321). Foulkes et al. (2005), in their study of child-directed speech among 39 mothers in North East England, found that phonetic variants used in this register differed markedly from inter-adult speech, and further noted effects with regard to the gender of the children in the dyads: speech to girls contained more standard variants than speech to boys, which contained higher rates of vernacular variants. This effect was more apparent for younger children in the sample.

### Studies of cross-cultural language input

Cross-culturally, children have different early language experiences, and the existing research on non-Western cultural groups indicates that there is a great deal of variation in the type and amount of direct language input children receive in the pre-school years (Hoff, 2006). Various studies have observed that in some non-Western cultures, young children are not seen as communicative partners, and receive very little child-directed speech as infants, in contrast to the patterns observed for North American and European caregivers. This been noted for Gusii mothers in Kenya (Richman et al., 1992; LeVine, 2004), Gapuners in Papua New Guinea (Kulick, 1992), the Kaluli in Papua New Guinea (Ochs and Schieffelin, 1994), Samoans in Western Samoa (Ochs and Schieffelin, 1994), the Warlpiri of central Australia (Bavin, 1992), K'iche' Maya speakers in Guatemala (Pye, 1992), Tzeltal speakers in Mexico (Brown, 2001), Javanese speakers in East Java (Smith-Hefner, 1998), and African-Americans in South Carolina (Heath, 1983). In many of these studies (e.g., Ochs and Schieffelin, 1994), the researchers suggest that a large amount of early language learning must be based on overheard, rather than child-directed, language, and that minimal direct speech to children may correlate with relatively late language development (Brown, 2001).

However, there is very little research describing language input in these non-Western societies on occasions when input *is* provided by caregivers, and even less looking at child language development in relation to input, beyond the body of research on the acquisition of language-specific grammatical and phonological features. In Indigenous Australia, a number of researchers have observed the use of “baby talk” registers (Laughren, 1984; Lee, 1987; Bavin, 1992, 1993; Kral and Ellis, 2008; Jones and Meakins, 2013; Turpin et al., 2014). This register is typically characterized by such features as phonological simplification, semantic simplification (for some groups of vocabulary items), repetition, slower speech rate and falling prosodic contour.

Outside the Australian context, Harkness' (1977) cross-sectional study found that Kipsigis caregivers in Kenya modified the length and complexity of their utterances as a function of their child interlocutor's MLU, adjusting their speech to the developmental stage of the child, leading Harkness to observe that “mothers and children in cultures far removed from our own modify their speech to children learning to talk in the same way that Americans do” (1977: 315). In contrast, there are claims that when caregivers in some other cultures talk to children, they do not use “baby talk”; for example, Ochs and Schieffelin (1994) observe that Kaluli and Samoan caregivers, though they may use pitch manipulation, do not engage in the morphosyntactic simplification synonymous with child-directed speech. Despite this, “Kaluli and Samoan children become fluent speakers within the range of normal developmental variation” (Ochs and Schieffelin, 1994, p. 494). Crago et al. (1997) have similarly found that of the minimal language input received by children in Inuktitut-speaking communities in Quebec, very little is morphosyntactically modified, but the children still achieve the major language development milestones at ages comparable to those for Western children. In reviewing the literature on child-directed speech, Lieven (1994) argues that the child-centered style of speaking may be one way of enabling children to learn and use their mother tongue, “but it is clearly not essential” (1994: 72), and concludes that children worldwide tend to learn language at around the same time, despite the diverse ways of speaking to (and around) children.

A further important aspect of the linguistic socialization of Indigenous children in Australia pertains to the high level of input children receive from interactants other than adults. In many Indigenous communities, children spend a great deal of their time playing and interacting with other children, with older children often taking on caregiving roles. Hamilton's (1981) observations in Arnhem Land describe how from the age of two, children are absorbed into a peer group of related children for support and learning experiences. These groups may develop into more structured “kid mobs” common for children from 5 to 9 years of age, resulting in less frequent interaction with adults. After-school and weekend activities may take place in kid mob groupings, which are largely determined by common linguistic and kinship ties. The linguistic consequences may be significant, with the “kid mob” a potentially dominant force in community language shift whereby younger speakers may even socialize adults in language choice (see Luykx, 2005 and Gafaranga, 2010). O'Shannessy (2012, 2013) has shown that children can play a significant role in language change, partly due to their spending time in peer groups, and partly due to the kind of input they receive. Loakes et al. (2013) addressed this aspect of community language shift in Yakanarra by investigating the input received from different-aged interlocutors with regard to code choice and found that less traditional language is found in children's interactions with younger interlocutors. These young interlocutors also use markedly more talk than older interactants.

While there is some research into normative language development for non middle-class, non-Western children, this area remains poorly understood, particularly in situations where the children grow up in multilingual communities. Research suggests that bi- or multilingual children progress through linguistic developmental stages at similar rates to monolingual children (Pearson et al., 1993; Paradis and Genesee, 1996; De Houwer, 2005), and in particular that when *all* the child's linguistic resources are considered (i.e., their knowledge of both/all languages), multilingual children have similar vocabulary sizes and grammatical abilities to monolingual children (Pearson and Fernandez, 1994; Nicoladis and Genesee, 1997; Patterson, 1998; Patterson and Pearson, 2004). However, in assessing children's abilities in each separate language, some research suggests that at comparable ages, bi- or multilingual children may have less developed vocabularies and grammatical abilities in each individual language than monolingual children in their sole language (Bialystok and Feng, 2011; Hoff et al., 2012). Other research indicates that language development progresses language-specifically (Marchman et al., 2004; Conboy and Thal, 2006). Language development for multilingual children is also clearly tied in with the issue of language dominance, which in turn is closely related to the type and quantity of language input children receive from carers (Lanza, 1997; De Houwer, 1999, 2007; Hoff et al., 2012). As for other areas of acquisition research, knowledge about language socialization in multilingual families tends to be based on research in Western cultures. Some research has investigated language socialization in language contact settings (Garrett and Baquedano-López, 2002; Luykx, 2005; Makihara, 2005; Gafaranga, 2010; O'Shannessy, 2011) including creole contexts (Garrett, 2005; Paugh, 2005), and it is clear that these non-Western contexts encompass an extremely diverse range of language learning environments, with many unique and context-specific factors that need to be considered in investigations of child language development.

We cannot assume that current understandings of child language input and language development hold true for children in linguistically diverse non-Western settings, and there is still a large amount of research to be done on the nature of the language received and produced by children in these settings, as well as the language development patterns that are normal for them. As Saxton (2009, 2010) points out, many non-Western language socialization studies are anthropological in nature, rather than designed specifically to investigate child language input and acquisition, particularly those cited as evidence of minimal or non-modified child directed speech.

There is evidently a need for targeted exploration of caregiver input behavior and child language production cross-culturally and cross-linguistically, and the present study contributes to this by providing input and production data for two Indigenous Australian communities: Yakanarra in the Kimberley area of Western Australia, and Tennant Creek in the Barkly region of the Northern Territory. The study focuses on language input by primary caregivers to a group of preschool children, and also focuses on the children's productive language. We use longitudinal data from two time-points, approximately 2 years apart, to describe changes in adult input over time and developmental patterns in the children's speech.

## Profiles of the communities

Both communities in this study are located in remote desert regions of Australia. Yakanarra is in the far north of the state of Western Australia, and Tennant Creek is centrally located in the Northern Territory.

### Yakanarra

Yakanarra consists of about 30 houses 110 km south-east of Fitzroy Crossing in the Kimberley region. Yakanarra is typical of a remote rural Indigenous community in Australia, with the majority of residents being Indigenous, and the few non-Indigenous residents working in the school, community center, or shop. Yakanarra was established in 1989 by Walmajarri people, the oldest of whom had left their traditional country and hunter-gatherer way of life in the 1950s.

The traditional language of Yakanarra is Walmajarri, but by 2006, when Moses was carrying out fieldwork, FVKriol had displaced Walmajarri as the language of everyday talk, although Walmajarri was still spoken to some degree by the older members of the community (see especially Loakes et al., 2013). Some Standard Australian English is spoken in limited circumstances, typically in formal contexts, and with non-Indigenous people (see e.g., Wigglesworth and Simpson, 2008, p. 20).

### Tennant creek

Tennant Creek is a remote, urban township in the Barkly region of Central Australia. It is a small town with a population of approximately 3000, half of whom are Indigenous. The township is located on Warumungu country, the traditional language of the area. The local Kriol variety, Wumpurrarni English (see Disbray, 2009), is the main language of everyday communication for most Warumungu people. This local variety shares features with other Kriol varieties spoken in Indigenous Australia, but has a number of local features, including the use of Warumungu-source features, such as insertional code-switches and semantic case-marking (Morrison and Disbray, 2008).

Like many contact settings, there is substantial variation in the way people in Tennant Creek use the contact variety Wumpurrarni English, with the speech varieties best understood as occupying a continuum from “lighter” or more acrolectal to “heavier” or more basilectal styles. Similarly to the situation in Yakanarra, Warumungu has undergone significant language shift, and is spoken as a full code only by a small number of elders, with younger adult speakers tending to be partial speakers (Morrison and Disbray, 2008).

### Comparing yakanarra and tennant creek

Yakanarra and Tennant Creek are both located in desert regions of Australia. Yakanarra is a remote and relatively closed community with little outside influence, while Tennant Creek is a larger town on a major highway, with a greater variety of people from varied language backgrounds.

## Aims

As discussed earlier, our previous work in both Yakanarra and Tennant Creek has shown that language shift is occurring rapidly, and children hear a variety of different input codes, with FVKriol in Yakanarra and Wumpurrani English in Tennant Creek being the main codes used. The input they hear varies along a continuum from more acrolectal to more basilectal determined by various factors including the person, the setting and the interlocutor. This study therefore examines both features of input to the children, and features of the production of the children at two points in time 2 years apart, when the children were approximately two and four.

The specific research questions we address are:

1. What are the characteristics of the children's language use at ages two and four in terms of MLT and MLU?
2. What are the characteristics of the caregivers' language use at the two time points in terms of MLT and MLU?
3. In these two communities, what kinds of multilingual practices can be observed in the children's and the caregivers' language use?

While analysis of Time 1 compared to Time 2 is sometimes considered a gross measure of development (c.f. Snow, 1995), we argue that it is a crucial first step to understanding longitudinal changes in child language in these communities. Given the lack of understanding about normative language development in Indigenous Australian communities generally, and particularly in areas undergoing rapid language shift, we hope that results of the current study will be used as a reference sample for other researchers working in this area.

In general, ongoing work in Indigenous Australian communities is largely a response to the fact that language acquisition in monolingual (especially English-speaking) communities is well-understood, yet language acquisition in multilingual Indigenous societies such as those in Australia is understudied (see for example the discussion in Wigglesworth and Simpson, 2008, p. 14). Additionally, it is a response to the need for basic, language-specific work on developmental patterns before a full theory of child language acquisition can be attained (see e.g., Slobin, 1997).

## Method

### Participants

For both communities there are four child participants in the study, as well as their main caregiver, who were their mothers in all cases but one—BM is Belinda's great-grandmother^5^. This gives four interactional pairs and eight participants in each community, with sixteen participants in total. Information about the participants is shown in Table 1 below. This includes pseudonyms which refer to the children, their sex, the code for their caregiver, the children's ages at Time 1 and 2, and the age difference across the time-points. The caregiver code corresponds to the first initial of the child and the initial “M” for mother, so for example the mother of the first child in the table, Natalie, is referred to as NM.

**Table 1:** **Participant details**.

**Community**	**Child**	**Sex**	**Caregiver**	**Child age time 1**	**Child age time 2**	**Time 2-time 1**
Tennant creek	Natalie	F	NM	2;7	4;7	2;0
Tennant creek	Melanie	F	MM	2;7	4;3	1;8
Tennant creek	Sarah	F	SM	1;8	3;7	1;11
Tennant creek	Belinda	F	BM	2;2	4;2	2;0
Yakanarra	Katherine	F	KM	3;6	5;6	2;0
Yakanarra	Olivia	F	OM	2;7	4;7	2;0
Yakanarra	Andrew	M	AM	2;2	4;7	2;5
Yakanarra	Emily	F	EM	2;6	4;6	2;0

All participants were female with the exception of Andrew in Yakanarra. At Time 1, they ranged in age from 1;8 to 3;6, and at time 2 from 3;7 to 5;6. The age range between the two time periods varied from 1;8 to 2;5, (average 2;0) with five children having exactly 2 years between the two times. The average age of the children in Tennant Creek at time 1 was 2;3 and 4;2 at Time 2; in Yakanarra it was 2;8 at time 1 and 4;10 at time 2. The caregivers range from being in their early twenties to early thirties, while BM is in her early seventies.

The fact that age is not balanced across the sessions is a limitation, but a reflection of the reality of data collection in Indigenous communities. In Indigenous Australia, people tend to be highly mobile. Communities are small and members are often traveling between different communities, visiting family and taking part in local events across a region. Following participants to record them in other locations is not feasible, given the remoteness of the communities and the distance that would need to be covered. As such, we have had to make compromises as far as exact comparability of child ages and difference between sessions is concerned. Furthermore, in Indigenous Australia, caregiving is shared by a range of older kin, including older children (i.e., the “kid mob” Hamilton, 1981; Andrews, 2008), grandparents and great grandparents and children therefore receive language input from a diverse group of speakers. To achieve maximal comparability, we have limited our study to four dyads in each location, and to child-caregiver interactions. This poses limitations to the breadth of representation of the range of participants and speech styles in such inherently dynamic speech settings. Despite this, we expect that results will be an important contribution to knowledge in the field of child language generally, with data from rarely studied locations which are undergoing rapid language shift, and where little is known about how children acquire language.

### Procedure

The corpus for this study comprises sixteen transcripts transcribed into CLAN^6^. Transcripts were based on video and audio-recorded interactions between the eight focus children and their primary caregivers, at the two different time periods. All transcripts are of equal length, with 100 lines being chosen for commensurability. Participants were primarily engaged in two types of tasks: prompted play (for example using toy cash registers, a doctor's kit or building with wooden blocks) and picture book-prompted story telling (Egan, 1986; O'Shannessy, 2004).

The data are a subset of materials collected in the first phase of a longitudinal study, The Australian Child Language Acquisition project (ACLA)^7^, now in its second phase. The first phase of the project focused on caregiver input to young Indigenous children, who were between the ages of 18 months and two and a half years at the beginning of the project. Language data were collected from children and caregivers every 6-months over a 4-year period. While data were collected in three remote Australian communities, this paper focuses only on Yakanarra and Tennant Creek.

In the transcripts, each morpheme was coded as either the local contact variety, or as the traditional language. It was not always straightforward to determine which words have been incorporated as loan words (and so should be coded as Kriol), and which are simply the product of code-switching (and so should be coded as traditional language). This is in part because no definitive description of the Kriol lexicon exists in either location. However, we were able to consistently apply our analysis across the data, with work on the Kriol lexicon in Yakanarra by Hudson (1985), and with Sandefur (1979) and Lee (2004) guiding our transcription, glossing and, in some areas, description of both the Tennant Creek and Yakanarra data. Words were coded as traditional language when the phonological and semantic structure matched that of the traditional language. All other cases were coded as the contact variety. In the extracts given throughout this paper, tokens identified as Walmajarri or Warumungu are underlined. Standard English, while certainly used in some instances in these communities, was not found in the child-caregiver interactions that we recorded.

For this study, we have collapsed acrolectal and basilectal Kriol forms into one category, despite the fact that speakers vary considerably along a continuum with respect to this. Separating out these varieties is, in practice, next to impossible as many elements are shared across the continuum and variation occurs in relation to interlocutor and topic as well as age and idiolect. Since distinguishing Kriol varieties is not a central concern of this study, we have not attempted such a categorization. While the resultant procedure is broad, and potentially limiting in terms of over-representing the traditional language, this exact coding procedure worked well for the Yakanarra data analyzed by Loakes et al. (2013). In that study, we were able to demonstrate distinct differences across age-groups in terms of language use with the contact variety, FVKriol, used as the main language by all speakers when interacting with young children. However, the traditional language Walmajarri, while rarely used by younger people, was used about a third of the time by speakers over the age of 50.

The following quantitative measures were used:

- Mean length of turn (MLT)
- MLT ratio
- Mean length of utterance (MLU)

This battery of measures illustrates the overall utterance length (MLT), conversational load (MLT ratio) and sentence complexity (MLU). Mean length of turn is the ratio of words to turn, where a turn is “a sequence of utterances spoken by a single speaker” (McWhinney, 2012, p. 92). MLT ratio is a related measure, calculated by dividing the focus child MLT over interlocutor MLT. Where the value is below 1.0 the interactant has a greater conversational burden, and greater than 1.0 means the child has a greater share (see McWhinney, 2012, p. 40).

Mean length of utterance is a measure of morphosyntactic complexity and consists of the ratio of morphemes to utterances, where an utterance can range from a single token to a full clause. MLU has not been without its critics, but has been widely used to measure children's language development despite criticisms relating to its lack of sensitivity to social context, and the unrepresentativeness of the early populations sampled by psycholinguists (e.g., Geneshi and Glupczynski, 2006). As one of a battery of measures though, it remains a valid technique.

While we acknowledge that these measures are limited in their depth of analysis, the approach has been chosen to provide an initial comparison of language use to and by children in the communities. It is by no means exhaustive. But to date, there has been no quantitative, longitudinal study of children's language development in these indigenous communities: this study is, thus, a first step in that direction.

## Results

### Language use

In both communities, the local Kriol variety was used overwhelmingly at T1 and T2 by both children and caregivers—well over 90% of morphemes. The exceptions were at T1 where Belinda used the Warumungu words *karnanti* (“mother”), *kampaju* (“father”), and *kupunta* (“burn”), repeatedly, and Melanie uttered only four morphemes, one of which was in Warumungu. Belinda's great-grandmother, BM, is a fluent Warumungu speaker who uses Warumungu frequently, including in her interactions with children. Similarly in Yakanarra, children and their caregivers use the traditional language at very low rates overall, although there are small increases in its use between T1 and T2. This is commensurate with our findings in Yakanarra (i.e., Loakes et al., 2013), where we saw that the older people in the community were more likely to use the traditional language in their interactions with children than younger adults were, as a result of which children used more traditional language in response. Similarly children sometimes responded entirely in Kriol when spoken to by older people entirely in the traditional language (Loakes et al., 2013)—a case of “receptive multilingualism” (see e.g., ten Thije and Zeevaert, 2007; ten Thije et al., 2012), or “two-way” conversation (Elwell, 1982). Meakins (2008) made similar observations in Kalkaringi for Gurindji and Gurindji Kriol.

A more detailed examination of the *nature* of the interaction between the codes may provide some insights about the ongoing pragmatic and interactional role of traditional languages. In Extract 1, where Melanie is looking at a picture book with her mother, the turn is predominantly in Wumpurrarni English, but with Warumungu (underlined) used occasionally. The Warumungu-usage which priviledges nouns (e.g., *julaka* “bird”) and case-markers (such as the possessive *–kayi*) is typical.

**Extract 1:** ***Melanie 4;3 and mother MM, T2***.


MM:   dat   lil    julaka   bin   go   na    im-kayi   mami   na
      DET   little bird     PST   go   LOC   3S POSS   mother now
      *the little bird went to its mother*.

MM:   yu   luk    deya
      2S   look   there
       *see there*.

MM:   im-kayi   mami   i    bin   kraiin   fo    im
      3S POSS   mother 3S   PST   cry      for   3S
      *his mother was crying for him*.

MM:   i    bin   ran   wai    dumuj     i    bin   git   los
      3S   PST   run   away   because   3S   PST   get   lost
      *he ran away from because he got lost*.


Similarly, in Yakanarra, in Extract 2 Olivia and her mother are sharing a book and code-switching between FVKriol and Walmajarri (underlined).

**Extract 2: *Olivia 2;7 and mother OM*, T1**.


OM:       hu   dijan?
          Who   this one
          *Who's this one?*

Olivia:   pirla
          *ghost*

OM:       na   parri   det   parri   parri   parri
          No   boy     DEM   boy     boy     boy
          *no boy, that's a boy, boy, boy*.

Olivia:   ei   parri
          Yes  boy
          *yeah boy*


Again, it is typically nouns that elicit Walmajarri. Indeed, Loakes et al. (2012) demonstrated that children in Yakanarra have a relatively good receptive knowledge of Walmajarri nouns related to the presence of these words in their input. Nouns are not the only targets of switching however. The traditional language is also used frequently for tag questions (e.g., *payi* “is that OK?” as in Extract 6 below). And in the following example, Emily's mother (EM) issues her command first in Walmajarri and then in Kriol. This use of both languages in parallel utterances has also been documented in Tennant Creek speech styles (Disbray, 2008):

**Extract 3:** ***EM's direction to Emily* 2;6, T1**.


EM:   yutanti
      Sit-down-IMP
      *sit down*

EM:   sidan
      Sit down
      *sit down*.


Receptive multilingualism, i.e., the use of one language per participant in an interaction is known to be a common practice in these communities (see e.g., the example in Loakes et al. (2013, p. 700) where a child speaks FVKriol to her grandmother and is responded to in Walmajarri). In Yakanarra it appears to be largely an intergenerational practice, and so perhaps is a product of language shift, but this is not the case in all communities (e.g., in Maningrida, Arnhem Land—see Elwell, 1982). Indeed, this kind of code-switching is known to have been a stable linguistic practice traditionally with distinct pragmatic and social functions (Wilkins and Nash, 2008; Singer and Harris, forthcoming).

Code-switching raises a number of issues and questions for our analysis and for research of this kind more generally. Firstly, it presents a challenge for data analysis in that while it is uncontroversial in theory that code-switching differs from lexical borrowing (i.e., where a foreign word has been fully adapted into a host language), it is not always clear in practice (Poplack, 2004). For example, if the surface form is uninflected, if it occupies a slot shared by both languages, and/or if phonological cues are absent or unclear, the task is by no means a straightforward one. The challenge is mitigated to some extent by the fact that the researchers know the communities well and are able to make judgments based on their knowledge of the standard adult language and common community linguistic practices. Furthermore, the methodological issue begs a larger question regarding the social reality of code distinction: are speakers attending to the difference between codes? If not, should the researcher?^8^ Of course in cases of multilingual language acquisition there is the added complication that young children may not yet have learnt to discriminate between codes as the adult target does. This is not a question we will dwell on here, but it is important to acknowledge that the necessity of distinguishing codes for the purpose of quantitative analysis can obfuscate the social reality in terms of how speakers actually understand and manipulate codes.

As we have seen, the contact varieties of Wumpurrarni English and FVKriol are the preferred languages for communication, but the traditional languages, Warumungu and Walmajarri, have a continued presence in interaction through a range of multilingual practices. Both communities appear to demonstrate similar uses of the traditional language in terms of the linguistic interaction of codes. However, an understanding of the social aspects of code use will require further research. In the next section we focus on *how* children's productive language develops across the time periods.

## Developmental measures

### Mean length of turn (MLT)

The MLT provides an impression of overall utterance length, while the related measure, MLT ratio, reflects the conversational load of each interactant. MLT is the average of the number of words used per turn, while conversational load is the ratio of child MLT over caregiver MLT.

MLT values and the associated MLT ratios are shown in Tables 2, 3 below for Tennant Creek.

**Table 2:** **MLT observations for focus children and caregivers, Tennant Creek**.

**Tennant Creek**	**Child (T1)**	**Child (T2)**	**CG (T1)**	**CG (T2)**
Natalie and NM	2.0	1.4	4.1	24.6
Melanie and MM	2.0	4.3	6.3	26.3
Sarah and SM	1.8	5.5	6.6	35.3
Belinda and BM	1.9	5.2	8.3	15.1
Average	1.9	4.1	6.3	25.3

**Table 3:** **MLT ratios, Tennant Creek**.

	**Time 1**	**Time 2**
Natalie and NM	0.48	0.06
Melanie and MM	0.32	0.16
Sarah and SM	0.27	0.16
Belinda and BM	0.23	0.35
Average	0.33	0.18

As might be expected, the Tennant Creek children use slightly more than double the number of words per turn at T2 compared to T1. At T1 the children all appear to be at a similar stage, with results ranging from 1.8 to 2.0 words per turn on average. At Time 2, all of the children, except Natalie, have a marked increase in words usage, ranging from 4.3 to 5.5 words per turn. And as we see below, Natalie's results may be task based.

For the caregivers, MLT values are always higher than the children's, especially at Time 2. At Time 1, the average MLT was 6.3 with a range of 4.1–8.3. However, the most striking result from this table is the average length of utterances used by the caregivers at Time 2, with an average of 25.3 words per turn, equating to four times as many words per turn on average than used at Time 1. Results for individual speakers range from 15.1 for Belinda's great-grandmother to 35.3 for Sarah's mother.

Extract 4 is from Sarah's mother at T2 and clearly shows the relatively monologic nature of her talk. She is telling a story and is not often interrupted by Sarah. This type of narration routine was observed in a previous study of Tennant Creek child-caregiver interaction (Disbray, 2008). Here adults tended to tell “elaborate” stories possibly as a strategy to maintain the children's attention and to model story-telling.

**Extract 4: *SM's interaction with Sarah 3;6, Time 2*.**


SM:      iya   damob        bin   jeis-im    na    luk
         Here  that group   PST   chase-TR   now   look
         *here, that group were chasing it, now look*.

SM:      dubala  bin   jeis-im   bat    im   na
         2 NOM   PST   chase-TR  QUANT^9^ 3S   now
         *two of them were chasing it now*.

Sarah:   na   na   dat   dat
         Now  now  DEM   DEM
         *now now, that that*.

SM:      stik   bin   pok-im   im
         Stick  PST   poke-TR  3S
         *the stick poked him*.

SM:      luk    what   kain   pants
         Look   what   kind   pants
         *look what kind of pants*.

SM:      stik   bin   pok-im    im
         Stick  PST   poke-TR   3S
         *the stick poked him*.

SM:      im,   gad,   im   still   gad   shangayi
         3S    got    3S   still   got   shanghai
         *he's got, he's still got the shanghai (sling-shot)*.

SM:      wan   bala   jeis-im
         One   3NOM   chase-TR
         *one of them chases it*.

SM:      deya   im-kayi   mami     na
         There  3S POSS   mother   now
         *there's it's     mother   now*


In Extract 5, Natalie is playing with blocks, and her mother provides a series of requests and explanations. She draws the child's attention to the construction depicted on the box containing the blocks, encouraging the child to build a similar construction. The especially long number of words per turn is typical of this session, yet markedly different to the length of utterance for the same speaker at T1. In this particular case, NM uses 32 words before Natalie's one word response.

**Extract 5: *NM's interaction with Natalie 4;7, Time 2*.**


NM:         yu   gid   dat   pitja   deya   luk
            2S   get   Dem   picture there  look
            *you get that picture there, look*.

NM:         si   deya
            See  there
            *see there*.

NM:         yu   trai   du   dat
            2S   try    do   that
            *you try to do that*

NM:         luk,   dem   deya
            Look   3Pl   there
            *look, them there*.

NM:         yu   kin   meik   haus
            2S   can   make   house
            *you can make a house*.

NM:         kasel
            castle
            *castle*.

NM:         si   tivi   an    bed   yu   kin   meik   im
            See  TV     and   bed   2S   can   make   3S
            *see the TV and bed, you can make them*

NM:         kwik
            quickly
            *quickly*.

NM:         weya    dat    Harvey?
            Where   that   Harvey
            *where's that Harvey?*

Natalie:    dawan
            That one
            *that one*.


The related MLT ratio (child/caregiver MLT) or conversational load is one way of representing the proportion of the utterance attributable to each speaker. We can expect this to be low at T2 given the long caregiver utterances as shown in Table 3,^10^.

These data suggest the conversational burden rests mostly with the caregivers in all instances, although T2 is more variable than T1 with a low MLT for Natalie and her mother, reflective of Natalie's minimal talk during the wooden block play, as seen in Extract 5 above. Melanie and Sarah have the same MLT ratio at T2 and Belinda is the only child to take on more of the conversational load at T2 compared to T1 increasing from 0.23 to 0.35 (still relatively low).

The picture at Yakanarra is different, with more variability amongst participants, as shown in Table 4 which presents the MLT.

**Table 4:** **MLT observations for focus children and caregivers, Yakanarra**.

	**Child (T1)**	**Child (T2)**	**CG (T1)**	**CG (T2)**
Katherine and KM	5.3	2.5	10.2	7.6
Olivia and OM	3.1	5.2	7.6	8.9
Andrew and AM	2.3	5.1	27.3	14.8
Emily and EM	4.7	4.7	5.1	3.3
Average	3.9	4.4	12.6	8.7

For the Yakanarra child participants, there is little difference between T1 and T2, on average. At T1, their average MLT is around double the average of the Tennant Creek children (at 1.9), and at T2 their MLT of 4.4 is only marginally higher than at Time 1, and than the Tennant Creek children's MLT at T2 (4.1). Thus, while Yakanarra children have a much higher average MLT at T1, by T2 they are very similar. It is likely that the MLT difference between Tennant Creek and Yakanarra children at T1 is linked to the difference in the average age between participants, since the Tennant Creek children are younger. At T1, the Yakanarra children's MLT values correlate with their age: Katherine is the oldest participant and has an MLT of 5.3, but Andrew, the youngest only averages 2.3. At Time 2 however, Katherine has the lowest MLT.

There is considerable variation in Yakanarra. Two children increase in MLT from T1 to T2 (Olivia and Andrew), and two do not—Katherine's MLT is lower at T2, while Emily's is the same. Thus, the range of values for MLT is quite broad at both times, between 2.3 and 5.3 words per turn at T1, and 2.5–5.2 at T2. This differs from the Tennant Creek results where the MLT fitted within a narrow range at each time period.

For the caregivers, results are also different from Tennant Creek. At T1, the Yakanarra children have twice the average words per turn than Tennant Creek children, and this is also true for caregivers, where the average 12.6 words per turn is double those for Tennant Creek. At T2, Yakanarra caregivers have a lower MLT than at T1, while in Tennant Creek they were four times higher. Additionally, MLT values for the Tennant Creek caregivers fall within a relatively narrow range of values, whereas for the Yakanarra caregivers there is a wide range from 5.1 to 27.3 at T1 and from 3.3 to 14.8 at T2. Finally, three Yakanarra caregivers have similar MLT values to their children; the exception is Andrew and AM, with AM having exceptionally high MLT values (27.3 at T1, and 14.8 at T12). This is reflected in the MLT ratio, as shown in Table 5.

**Table 5:** **MLT ratios, Yakanarra**.

	**Time 1**	**Time 2**
Katherine and KM	0.52	0.33
Olivia and OM	0.41	0.58
Andrew and AM	0.08	0.34
Emily and EM	0.92	1.42
Average	0.48	0.67

Yakanarra children have, on average, a higher MLT than the Tennant Creek children at both time periods. This indicates that the Yakanarra children generally have a greater share of the conversation when interacting with their caregivers compared to the Tennant Creek children in these sessions. While neither group is approaching an equal conversational load, the Yakanarra children have a higher MLT ratio at T1 than the Tennant Creek children had at both T1 and T2, and they increase their conversational share at T2.

A brief note about individual variation is important given these results. At T1, Andrew's ratio is especially low at 0.08 indicating that he barely contributes, verbally, to the interaction. Emily, on the other hand, has an MLT ratio approaching 1.0 suggesting equal conversational load between her and her mother. At T2, Katherine and Andrew have similar, relatively low, MLT ratios although this is an increase for Andrew, but a decrease for Katherine. Emily is the only participant with a higher conversational burden than her mother. Emily's input remains the same at T1 and T2, but her mother's changes, with less input at T2 (see Extract 10 for an example of this pair's interaction).

Extracts 6 and 7 from Olivia's interaction with OM are from an identical task where a book is being read. At T1, Olivia averaged three words per turn on average, and five at T2. OM averaged 7.6 words per turn at T1 and 9 at T2 yielding a slightly greater conversational load for Olivia at T2.

**Extract 6:** ***OM's interaction with Olivia 2;7, Time 1***.


OM:       en    wat    i    bin   du?
          And   what   3S   PST   do
          *And what has it done?*

OM:       luk    wat    i    bin   du?
          Look   what   3S   PST   do
          *look, what has it done?*

OM:       i    bin   fol    dan,   payi?
          3S   PST   fall   down   isn't it
          *It's fallen down, hasn't it?*

Olivia:   ye
          yes
          *yeah*.

OM:       oi   i    bin   fol    dan
          Oh   3S   PST   fall   down
          *oh, it's fallen down*.

Olivia:   no   i    rait.
          No   3S   right
          *no its OK*.

M:        en    we      det   jikjik    la    is   mami,    payi?
          And   where   the   chicken   LOC   3S   mother   isn't it
          *and where is that chicken's – its at his mother's isn't it?*

Olivia:   im   iya
          3S   here
          *it's here*.


At T2, the development in Olivia's interactional capability is clear. Here she has more, and longer turns, than at T1.

**Extract 7:** ***OM's interaction with Olivia 4;7, Time 2***.


OM:       en    we      dijan   fo    shanghai?
          And   where   Dem     for   shanghai
          *and where is the shanghai (sling-shot)*.

Olivia:   de
          there
          *there*

Olivia:   den    dei   tjeis-im   bat     im   tjeis
          Then   3Pl   chase-TR   QUANT   3S   chase
          *then they chase it, chase*

Olivia:   im   bat   im   tjeis-im   bat     im
          3S   but   3S   chase-TR   QUANT   3S
          *it, chased it*.

OM:       a    pupala
          Ah   poorthing
          *ah poor thing*.

Olivia:   ye
          yes
          *yeah*.

OM:       dei   bin   ged   im   hepi
          3PL   PST   get   3S   happy
          *They were happy*.

Olivia    la    nes    i    bin   bi
          LOC   nest   3S   PST   be
          *it was in the nest*

Olivia:   we      is     mami     wan?
          Where   POSS   mother   Det
          *where is its mother?*

Olivia:   de?
          there
          *there?*

OM:       na   is     mami     bin   go   ged-im
          No   POSS   mother   PST   go   get-TR
          *no its mother has gone*
          bat     mangarri   bla   im
          *QUANT*   food       for   him
          *to get food for it*.


Recall that Andrew is the youngest child in the Yakanarra cohort, and his interaction with his mother patterned somewhat differently to others. In Extract 8, they are looking at figurines, and the reasons for Andrew's comparatively low MLT (5.1 to AM's 14.8) as well as relatively low conversational load (0.38), are clear.

**Extract 8:** ***AM's interaction with Andrew 4;7, Time 2***.


AM:       ei    dis   da   mami     wan   fo    det   beibi
          Hey   Dem   is   mother   NOM   for   Dem   baby
          *hey this is the mother for that baby*,

          si    i    garra   bodul.
          See   3S   get     bottle
          *see it's got a bottle*.

Andrew:   hu   detwan?
          Who  that one
          *who's that?*

AM:       ai   dono         hu    det
          1S   don't know   who   Dem
          *I don't know who that is*.

Andrew:   xxx   ting.
          Xxx   thing
          *[unintelligible word] thing*

AM:       ye    lil      skul     gel
          Yes   little   school   girl
          *yeah it's a little school girl*.

AM:       en    diswan     god   lil      mobailfon
          And   this one   got   little   mobile phone
          *and this one has got a little mobile phone*.

AM:       yu   luk-im    lil      mobailfon
          2S   look-TR   little   mobile phone
          *you look, a little mobile phone*.

Andrew:   ye    ai   garram   iya
          Yes   1S   got      here
          *yeah I've got it here*.


In this section, we have seen that utterance length is used differently in each of the two communities analyzed. In Tennant Creek, both children and caregivers tend to have an increase in MLT over time. As illustrated, the caregivers use exceptionally long utterances at Time 2, and this impacts on conversational load where we saw that Tennant Creek children take on less of the overall conversational burden at Time 2. The Yakanarra caregivers, by contrast, use shorter utterances on average at Time 2, while the children have slightly longer utterances. In Yakanarra, the children's conversational burden is generally greater at Time 2, and one of the children actually has a higher conversational load than her caregiver.

The caregivers in the two communities seem to respond differently with respect to quantity of talk. Tennant Creek caregivers use more talk at Time 2 perhaps recognizing their children's increased receptive capacity, while the Yakanarra participants use less talk on average perhaps making room for their children's productive capacity. It may be the age difference of the children, with the children older at Yakanarra, that elicits this response.

### MLU

MLU is a measure of morphological complexity, reflecting the ratio of morphemes to utterances, and is generally considered useful for comparing development across time points. MLU is often compared to standardized values, but this is not possible here as there are no standardized MLU values for Kriol and the traditional languages featured. However, values can be used comparatively across the corpus. In this study, MLU is measured using morphemes primarily from Kriol, with some traditional language morphemes also included in the analysis. This is justified because, as seen earlier, the number of traditional language morphemes used by participants was very minimal. MLU values for Tennant Creek are presented in Table 6 below.

**Table 6:** **MLU, Tennant Creek**.

	**Child (T1)**	**Child (T2)**	**CG (T1)**	**CG (T2)**
Natalie and NM	1.8	3.7	1.4	3.5
Melanie and MM	2	6.3	3.9	7.5
Sarah and SM	1.5	5.4	4	5.6
Belinda and BM	1.6	5.9	4.1	5.9
Average	1.7	5.3	3.4	5.6

All children and their caregivers use morphologically more complex utterances at T2 with the children having the greatest increase from 1.7 to 5.3. At T2, both groups have a similar average MLU (5.3 and 5.6), reflecting similar grammatical complexity despite the marked differences in utterance length described earlier. At T1 the caregivers tend to have a much higher MLU than their children (besides Natalie and NM), but at T2 they are almost at parity. The exception is Melanie and MM, with MM having a higher MLU than Melanie.

As with MLT, the Tennant Creek data again falls within a relatively narrow range, although Natalie and NM have somewhat lower MLUs, and Melanie and MM slightly higher values. Extract 5 gave an example of Natalie and NM's interaction at T2 and Extract 9 is an example from Melanie and MM's (see also Extract 1). Verbal morphemes (preceded by an underscore) help to explain the higher MLU. Both participants use relatively long utterances providing a useful illustration of average turn length.

**Extract 9:** ***MM's interaction with Melanie 4;3, Time 2***.


MM:        an    dei   bin   keriy   im,
           And   3Pl   Pst   carry   3S
           *and they were carrying it*,

           keriy-im   *bat*     im   na     dat   nes,
           carry-TR   QUANT   3S   from   Dem   nest
           *carrying it from that nest –*

           i    bin   jamp   of    fom    dat   nes
           3S   Pst   jump   off   from   Dem   nest
           *it jumped off from that nest*

Melanie:   an    dei   gada     gid   im   na   ini?
           And   3Pl   got to   get   3S   now  isn't it
           *and they have to get it now, don't they?*


Yakanarra results for MLU are shown below.

In Yakanarra, MLU is narrower than Tennant Creek between 2.4 and 4.4, with caregivers having a higher MLU than the children at both times. Emily and EM are the exception to this, with Emily's MLU at T2 slightly higher than EM's. MLU increases at T2 for the children, and decreases slightly for the caregivers. Individual variation is fairly minimal as with the other measures analyzed in this study.

Average values point to an increase in child MLU across the time periods, with three of the four children showing higher MLU values at Time 2 (an increase of 1.2 in each case). Katherine is the exception here, with a slightly higher MLU at Time 1 (3.0 compared with 2.6). For the caregivers, average MLU decreased slightly across the time periods. Results in Table 7 show that this is the case for all except OM, who has a slightly higher MLU at Time 2 (4.4 compared with 5.0).

**Table 7:** **MLU, Yakanarra**.

	**Child (T1)**	**Child (T2)**	**CG (T1)**	**CG (T2)**
Katherine and KM	3	2.6	5.1	4.4
Olivia and OM	2.6	3.8	4.4	5
Andrew and AM	2.1	3.3	4.8	4.3
Emily and EM	2	3.2	3.1	2.7
Average	2.4	3.2	4.4	4.1

Examples of Olivia (and OM's) speech are given in extracts 2, 6, and 7. Extracts 6 (T1) and 7 (T2) in particular illustrate Olivia's increase in morpho-syntactic complexity across the time periods. Extract 10 shows Emily interacting with EM, which demonstrates EM's relatively low MLU T2, given the demonstrably low morpho-syntactic complexity of her turn (it is also indicative of the pair's MLT ratio, as discussed earlier).

**Extract 10:** ***EM's interaction with Emily 4;6, Time 2***.


Emily:   en    de    det   beed
         And   Dem   Dem   bird
         *and there, that bird*.

EM:      yu    luk    det   nes
         2Pl   look   Dem   next
         *you look, that nest*.

Emily    en    det   nes
         And   Dem   nest
         *and that nest*.

Emily:   en    de      i    flaiing
         And   there   3S   flying
         *and there it's flying*.


These MLU results indicate that in both communities the children use grammatically more complex utterances at T2. At both times, caregivers use more complex utterances than their children, but in Yakanarra the caregivers use less complex utterances at T2 than at T1. In other words, while children's language is developing over the 2-year period, as would be expected, caregiver responses pattern differently in each community.

## Discussion and conclusion

This study compared language use in two remote Indigenous Australian communities. Reflecting a loss of the traditional languages of the area, creole varieties—Wumpurrarni English and Fitzroy Valley Kriol, respectively—are used predominantly by the child-caregiver pairs studied in Tennant Creek and Yakanarra. The individual variation in language use observed was consistent with our previous study in Yakanarra where traditional language tends to be reserved for use by, and with, older participants aged 50+ (e.g., Loakes et al., 2013). McConvell (2008) also discusses a general trend for language shift in Indigenous Australia, where children appear to be largely monolingual (speaking a variety of Kriol). This compares to the situation previously in Australia, where community members tended to be multilingual in two or more traditional languages as well as English (e.g., Brandl and Walsh, 1982; Singer and Harris, forthcoming).

While our results point to a general decline in multilingualism and its attendant linguistic practices in Indigenous Australia, we have noted a range of uses for the surviving elements of the traditional languages featured. Code-switching and receptive multilingualism have important social and pragmatic functions, even, or perhaps especially, in cases of language change. Indeed, it can be the case that low-frequency linguistic forms carry significant pragmatic force (i.e., contributing to “markedness”). McConvell (2008) notes that such uses of traditional languages may have strong social meaning as “acts of identity” (see also Le Page and Tabouret-Keller, 1985).

As found in the early studies of caregiver talk (e.g., Snow, 1972, 1977; Sachs et al., 1976; Ferguson, 1977) for the caregivers in Tennant Creek, speech increases in MLT with age, reflecting similar patterns of development and we may assume, consequently, that caregivers make similar adjustments in terms of fine-tuning to their speech as those found in previous studies such as Vosoughi (2010) and that this is a function of the child's age. In contrast, the Yakanarra caregivers tend to use shorter and fewer turns at T2, and may respond to their children's language development by making room for their productive language capacity. For caregivers then, the main difference across the communities is the *quantity* of talk used.

In both Tennant Creek and Yakanarra we observed the general developmental characteristics of focus children using data collected approximately 2 years apart. The children displayed more developed language skills at T2, an unsurprising result given that they were on average 2 years older than at T1. While productive language capacity was not clear for Tennant Creek when we analyzed MLT and MLT ratio, we might infer that the especially long turns used by their caregivers is a response to their greater receptive knowledge at T2.

While not an explicit focus of this study, work on Baby Talk registers in Australian languages formed part of the theoretical grounding of register (see Laughren, 1984; Bavin, 1992, 1993; Kral and Ellis, 2008; O'Shannessy, 2011; Jones and Meakins, 2013; Turpin et al., 2014). We are, thus, able to reflect on the language input to the children in our study with this work in mind. Certain features noted in previous studies were observable in our data (largely use of repetition (see, e.g., Extract 2) and slower speech rate), but not the full range of features described in the studies above. Further research is required to fully investigate the existence and nature of this register in Tennant Creek and Yakanarra.

As discussed in the introduction, relatively little is known about child language development outside middle-class Western societies, and measures of analysis have tended to develop from languages spoken in these environments. As such, it is worth reflecting on our results to determine the validity of the measures we used for analysing contact varieties. One measure in particular, MLU, was used in this study in quite broadly and language types were grouped together (i.e., both Kriol and the traditional language) in calculating it. However, use of the traditional language was quantitatively minimal and so this is unlikely to have had much impact on results.

A crucial issue, which warrants further investigation, is that of variation. Heavy Kriol varieties have a more complex morphology and use grammatical and case-marking morphemes not used in English. At T1, where children ranged between 1;8 and 3;6, there was little evidence that they used such morphology. However, the children use a wider variety of verbal and nominal morphemes, and more of them, at T2 as reflected in the higher MLU for both cohorts. This could be for a number of reasons—the data may indicate acquisition of morphology, but it may also be an indication that children are using a heavier style of Kriol at T2. Semantic case-marking is also an optional feature, often occurring with relatively low frequency, so it may simply be that case-marking did not appear in the T1 data. In future work, it will be important for researchers to analyse individual speaker styles closely, and to relate them to children's stylistic use. Cross-sectional data from older children in Tennant Creek show use of complex morphology, through the use of transitive marking on verbs, and semantic case-marking (Disbray, 2009), particularly in heavy WE styles. These results are useful for contributing to knowledge on the types of values to expect for Kriol varieties where MLU is concerned, and a more detailed analysis, beyond the scope of this paper, may throw further light on this. The results of this study support those of Hoff (2006) who observed considerable variation in type and amount of direct language input children in these age groups received. These communities are undergoing, or have undergone in recent year, sizeable shifts in the languages spoken, commensurate with increasing variability in the input the children receive (see Loakes et al. (2013) for further discussion of this phenomenon).

This study has provided an initial insight into child-caregiver interaction in two remote Indigenous communities. Despite the different community structures—with Yakanarra being relatively closed, and Tennant Creek having more varied demographics—results are similar in terms of general developmental patterns, and the language types used. The creole variety local to each community is the preferred language of communication at both time points. For measures of morphosyntactic complexity (MLU), children in both communities use more complex utterances at T2 than at T1, and child-caregiver averages are similar to each other at T2. For measures of utterance length (MLT), there are more notable differences between the two communities for both children and caregivers at T2; for children in Tennant Creek, MLT doubled on average between T1 and T2, while in Yakanarra children were more variable. Strikingly, caregivers' MLT quadrupled between T1 and T2 in Tennant Creek, while in Yakanarra caregivers' MLT was lower at T2 than T1. We note, however, that the samples used here were convenience samples, and very small ones with only four dyads in each community. We may attribute some of the different results also to the fact that the children in Tennant Creek were younger at the start of the study than those in Yakanarra.

In tandem with Loakes et al.'s (2013) findings for Yakanarra, this research has contributed to a depiction of both language input to children, as well as their own language production. The former study elucidated how this language varies synchronically (according to interlocutor age), while the current study has focused on how children's language varies diachronically across time points 2 years apart. These results are a start in developing a picture of how children acquire language in these two multilingual Indigenous Australian communities.

### Conflict of interest statement

The authors declare that the research was conducted in the absence of any commercial or financial relationships that could be construed as a potential conflict of interest.

## Footnotes

^1^ In the Torres Strait Islands off Northern Queensland, the creole variety spoken is referred to as “Torres Strait Creole.” The spelling Kriol stems from the orthography developed for Roper River Kriol (Sandefur, 1979).

^2^ See Harris (1993) for a discussion of the development of Kriol in Australia, and more information about the varieties spoken.

^3^ http://languages-linguistics.unimelb.edu.au/past-projects/acla1

^4^ See Moses (2009), Disbray (2009), and Meakins (2011) respectively.

^5^ It is not unusual to have a great grandmother in an Indigenous community.

^6^ http://childes.psy.cmu.edu/

^7^ The first phase of this project is described in greater detail in Wigglesworth and Simpson(2008, see esp. pp. 19–27). The second phase of the project, currently underway, is investigating issues faced by Indigenous children as they enter the formal school system where they encounter Standard Australian English, of which they often have little knowledge, as well as significant cultural differences.

^8^ See work on code-mixing and “translanguaging” for further exploration of these points, especially (García, 2007; Creese and Blackledge, 2010; García and Wei, 2014).

^9^ “bat” is an event quantifier indicating the event is not performed once by one individual.

^10^ A ratio of 1 would indicate equality in conversational load, and any value less than 1 means the caregiver has a greater conversational burden than the child.
